# Beyond typical pathogens: pleural empyema caused by *Aggregatibacter aphrophilus*

**DOI:** 10.36416/1806-3756/e20260094

**Published:** 2026-09-14

**Authors:** Beatriz Dias, Ana Correia, Teresa Belo, Ana Filipa Matos, António Reis

**Affiliations:** 1. Serviço de Pneumologia, Unidade Local de Saúde Viseu-Dão Lafões, Viseu, Portugal

## TO THE EDITOR:

The acronym “HACEK” refers to a group of gram-negative bacilli that colonize the oral cavity and gastrointestinal tract and have the potential for invasive disease, abscess formation and endocarditis. *Aggregatibacter* (formerly *Haemophilus*) *aphrophilus* is part of this group.^1^ ^,^ ^2^

A 19-year-old male student had no history of chronic respiratory pathologies or other systems/organs, previous hospitalizations, regular medication use, drug allergies, recent trips, nor a history of poor oral hygiene, recent dental procedures, or periodontal infection. The patient presented to the emergency department with odynophagia and was diagnosed with bacterial tonsillitis, treated with amoxicillin/clavulanic acid, resulting in clinical resolution. Two months later, he re-presented with a two-week history of left-sided pleuritic chest pain, exacerbated by trunk movement and partially relieved by acetaminophen and muscle relaxants. There was no history of fever, cough, dyspnea, orthopnea, or paroxysmal nocturnal dyspnea. On evaluation, hypoxemic respiratory failure was noted. A chest X-ray demonstrated complete opacity of the left hemithorax (Figure 1A). Laboratory tests revealed leukocytosis with neutrophilia (44.27 × 10⁷/L; 93% neutrophils), as well as elevated C-reactive protein (35.99 mg/dL) and procalcitonin (1.8 ng/mL) levels. Chest CT showed a large left-sided pleural collection occupying nearly the entire hemithorax, with complete opacification of the left lower lobe and lingula, suggestive of atelectasis (Figure 1B). Chest ultrasound demonstrated a multiseptated left pleural effusion, a diagnostic thoracentesis was performed, and slightly cloudy liquid came out. The pleural fluid analysis was concordant with complicated effusion [pH = 6.86, glucose < 4 mg/dL, normal adenosine deaminase level, and 2,133 leukocytes/m^3^ (predominance of mononuclear cells)]. A left thoracic drain was placed. After blood cultures were taken and empirical antibiotic therapy was initiated with piperacillin/tazobactam and clindamycin. The patient was referred to the pulmonology team, with a favorable initial evolution with apyrexia on the third day of hospitalization. The drain was removed after seven days, due to non-functioning drainage. Given the highly organized nature of the pleural collection and the onset of fever on the following day after the drain was removed, pulmonary decortication via thoracotomy was performed on day 10 with no complications. Gram staining of the pleural fluid did not reveal microorganisms, but *A. aphrophilus* was isolated in cultures after seven days. No antimicrobial susceptibility testing was carried out. There was no microbiological growth in two sets of blood cultures, within a one week interval. Due to a new record of fever on day 15 of antibiotic therapy, treatment was escalated to meropenem use, completing a 4-week course of antibiotics, with clinical and radiological resolution.

**Figure 1:**
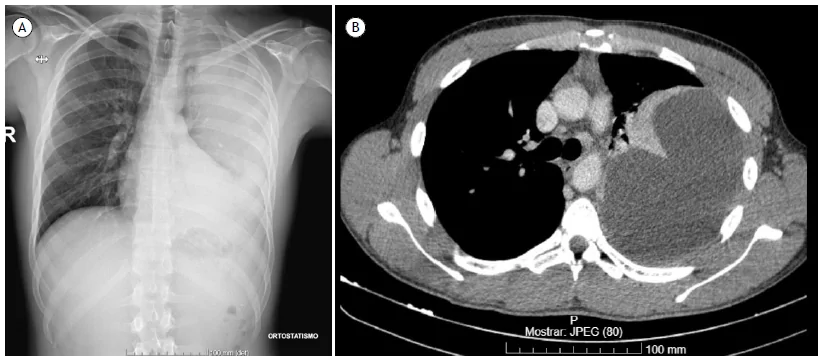
In A, chest X-ray on admission to the emergency room. In B, chest CT scan at admission showing slight deviation of the mediastinum to the left, large pleural effusion to the left, occupying almost the entire left hemithorax, and complete opacification of the left lower lobe and lingula (possible atelectasis).

On account of isolating an HACEK group bacilli, a transthoracic echocardiogram was carried out with normal results and no evidence of intracardiac infectious involvement. The rest of the study was negative for HIV, HBV, and HCV serologies. Given the favorable evolution after decortication and antibiotic therapy, the patient was discharged.

Pleuropulmonary infections with *A. aphrophilus* are rare. Since 1965, when the first described empyema caused by this microorganism was described, only six other cases have been reported, as summarized in Table 1. Some risk factors have been described, such as splenectomy, alcohol and drug abuse, smoking, and poor oral hygiene (cavities and missing dental pieces).^3^ ^-^ ^9^ However, in this case, the patient was not predisposed to these risk factors, implying that this bacterium has the potential to infect immunocompetent individuals.

**Table 1:** Clinical characteristics, imaging findings, management, and outcomes of reported pleuropulmonary infections caused by *A. aphrophilus*.

Case Report / Year	Age	Sex	Risk Factors	Clinical Presentation	Imaging Findings	Microbiological Diagnosis	Outcome
Capelli et al.^6^	49	Female	None identified	Dyspnea, cough, pain in the left scapular region (1 month)	Chest X-ray: large left pleural effusion with opacification	Pleural fluid culture	Clinical resolution after decortication and penicillin and tetracycline
Ratnayake et al.^5^	65	Female	None identified	Fever, dyspnea, dry cough (3 weeks)	Chest X-ray: right-sided pleural effusion with opacification	Pleural fluid culture	Recurrent empyema; eventual resolution after prolonged antibiotics (amoxicillin, doxycycline) and drainage
Rodriguez-Segade et al.^7^	49	Male	Smoking, alcohol use, poor oral hygiene/periodontitis	Fever, dyspnea, productive cough, constitutional symptoms (3 months)	Chest CT: right lung infiltrates with pleural effusion	Pleural fluid culture: *Aggregatibacter aphrophilus* + *Parvimonas micra*	Clinical improvement after antibiotics (linezolid + imipenem switch to amoxicillin + clavulanic acid after cultures) and pleural drainage
Belkacem et al.^9^	43	Male	Splenectomy; injection drug use	Fever, confusion, meningitis syndrome, and respiratory symptoms (2 months)	Brain MRI: multiple abscesses and ventriculitis; Chest CT: cavitating pneumonia with hilar adenopathy	16S rDNA PCR from brain abscess (cultures negative)	Clinical resolution after 6 weeks of cefotaxime
Sumer et al.^8^	33	Male	None identified	Fever, malaise, epigastric discomfort, nausea (10 days)	Abdominal US/CT: multiple hepatic abscesses; Chest CT: bilateral pulmonary nodules/abscesses	16S rDNA PCR from liver tissue (cultures negative)	Clinical and radiological resolution after 6 weeks of doxycycline
Lu et al.^3^	73	Male	None identified	Fever, right hip pain (6 days)	CT scan: psoas abscess, pulmonary infection, and pleural effusion,	Blood culture (pus and pleural fluid cultures negative)	ceftriaxone + levofloxacin, drainage of psoas abscess and pleural effusion.

Several pathophysiological mechanisms may explain the progression from oropharyngeal infection to pleural empyema in this patient. *A. aphrophilus* is a commensal organism of the oral cavity and upper respiratory tract, and aspiration of colonized oropharyngeal secretions represents a plausible route of infection, particularly following acute tonsillitis with increased bacterial burden. Transient, low-grade bacteremia is another possible mechanism, as HACEK organisms are known to cause intermittent bacteremia that may evade detection in routine blood cultures; therefore, the absence of bacteremia does not exclude hematogenous dissemination. A third possibility is an initially unrecognized pulmonary infection that subsequently progressed to complicated parapneumonic effusion and empyema. Previous reports of *A. aphrophilus* empyema and invasive infections have negative blood culture results similarly described, despite microbiological confirmation from sterile sites, supporting the interpretation that negative blood cultures in this context do not rule out transient bacteremia.^7^ Prior antibiotic exposure further reduces blood culture sensitivity. HACEK organisms are fastidious, slow-growing gram-negative bacilli that often require prolonged incubation and specific growth conditions. These characteristics explain why standard microbiological cultures usually remain negative. Consequently, abscess drainage is helpful for microbiological diagnosis by PCR-amplified 16S rDNA sequencing, especially for detection of the fastidious organism.^9^ Metagenomic next-generation sequencing (mNGS) represents an emerging diagnostic tool, particularly in culture-negative infections, particularly in infectious endocarditis.^10^ Unlike targeted approaches, mNGS enables unbiased detection of bacterial DNA, which may be advantageous for fastidious organisms such as HACEK species. However, its routine clinical use remains limited by cost, availability, and challenges in data interpretation.^10^

The predominance of mononuclear cells in the pleural fluid is atypical for acute bacterial empyema, which is usually neutrophil-predominant. However, this finding may be explained by the subacute disease course, prior antibiotic exposure, or a pathogen-specific inflammatory response. Similar pleural fluid profiles have been reported in subacute or partially treated pleural infections.

The optimal antibiotic treatment and duration for pleuropulmonary infection due to *A. aphrophilus* are not well established, and antimicrobial susceptibility testing was not performed due to technical limitations in routine testing of fastidious HACEK organisms at our institution, which was a limitation. Nevertheless, *A. aphrophilus* demonstrates relatively predictable susceptibility patterns based on published data, with consistent susceptibility to third-generation cephalosporins and fluoroquinolones, and variable susceptibility to ampicillin due to beta-lactamase production.^3^ ^,^ ^4^ ^,^ ^9^ Tetracyclines have also been reported as an adequate treatment for abscesses in other locations.^8^ Initial empirical therapy with piperacillin-tazobactam and clindamycin was selected to provide broad coverage for community-acquired pleural infection, including anaerobic oral flora, and antitoxin properties that may be beneficial in invasive streptococcal infections, since streptococcus is a common cause of tonsillitis. Clinical deterioration with recurrence of fever occurred despite this regimen, likely reflecting inadequate source control rather than antimicrobial failure. Following surgical decortication, antibiotic therapy was escalated due to persistent systemic inflammation. Antimicrobial decisions were therefore guided by established HACEK treatment recommendations and clinical response rather than isolate-specific susceptibility results and duration of antibiotic therapy according to recommendations of The British Thoracic Society guidelines on managing pleural infections, which recommend from two to six weeks of antibiotic therapy, according to clinical response.^11^

Although invasive infections caused by *A. aphrophilus* are uncommon, they have been repeatedly reported in young, immunocompetent individuals with no classical risk factors. This suggests that the host immune status alone does not fully account for disease development. Local factors such as mucosal disruption during tonsillitis, bacterial virulence traits, and delayed source control might have contributed to disease progression in this patient.

This case contributes to the limited literature on *A. aphrophilus* pleural empyema by illustrating disease progression following tonsillitis in a young immunocompetent individual, who presented with neither bacteremia nor endocarditis, showing atypical pleural fluid cellularity and failure of broad-spectrum antimicrobial therapy without adequate source control. It reinforces the importance of considering HACEK organisms in pleural infections with negative routine cultures and highlights the central role of surgical management in advanced empyema.
